# Circ_0026416 downregulation blocks the development of colorectal cancer through depleting MYO6 expression by enriching miR-545-3p

**DOI:** 10.1186/s12957-021-02407-y

**Published:** 2021-10-14

**Authors:** Lei Zhang, Ranran Yu, Chunhua Li, Yu Dang, Xiaoyu Yi, Lei Wang

**Affiliations:** 1 Department of General Surgery, The Second Affiliated Hospital of Shandong University of Traditional Chinese Medicine, Jingba Road, Shizhong District, Jinan City, 250001 Shandong Province China; 2 Department of Pathology, The Second Affiliated Hospital of Shandong University of Traditional Chinese Medicine, Jingba Road, Shizhong District, Jinan City, 250001 Shandong Province China; 3 Cancer Center, The Second Affiliated Hospital of Shandong University of Traditional Chinese Medicine, No.1 Jingba Road, Shizhong District, Jinan City, 250001 Shandong Province China

**Keywords:** circ_0026416, Colorectal cancer, miR-545-3p, MYO6

## Abstract

**Background:**

Emerging evidence reveals that the initiation and development of human cancers, including colorectal cancer (CRC), are associated with the deregulation of circular RNAs (circRNAs). Our study intended to disclose the role of circ_0026416 in the malignant behaviors of CRC.

**Methods:**

The detection for circ_0026416 expression, miR-545-3p expression, and myosin VI (MYO6) mRNA expression was performed using quantitative real-time PCR (qPCR). CCK-8 assay, colony formation assay, transwell assay, and flow cytometry assay were applied for functional analysis to monitor cell proliferation, migration, invasion, and apoptosis. The protein levels of MYO6 and epithelial mesenchymal-transition (EMT) markers were detected by western blot. Mouse models were used to determine the role of circ_0026416 in vivo. The potential relationship between miR-545-3p and circ_0026416 or MYO6 was verified by dual-luciferase reporter assay and RIP assay.

**Results:**

The expression of circ_0026416 was increased in CRC tumor tissues and cell lines. Circ_0026416 downregulation inhibited CRC cell proliferation, colony formation, migration, invasion, and EMT but induced cell apoptosis in vitro, and circ_0026416 knockdown also blocked tumor growth in vivo. MiR-545-3p was a target of circ_0026416, and rescue experiments indicated that circ_0026416 knockdown blocked CRC development by enriching miR-545-3p. In addition, miR-545-3p targeted MYO6 and inhibited MYO6 expression. MiR-545-3p enrichment suppressed CRC cell malignant behaviors by sequestering MYO6. Importantly, circ_0026416 knockdown depleted MYO6 expression by enriching miR-545-3p.

**Conclusion:**

Circ_0026416 downregulation blocked the development of CRC through depleting MYO6 expression by enriching miR-545-3p.

**Highlights:**

1. Circ_0026416 downregulation inhibits CRC development in vitro and in vivo.
2. Circ_0026416 regulates the expression of MYO6 by targeting miR-545-3p.
3. Circ_0026416 governs the miR-545-3p/MYO6 axis to regulate CRC progression.

**Supplementary Information:**

The online version contains supplementary material available at 10.1186/s12957-021-02407-y.

## Introduction

Colorectal cancer (CRC) has an increasing incidence in recent years, mainly due to poor diet, poor lifestyle, smoking, and physical inactivity [1]. CRC has been the third most common cancer in 2020, accounting for 10% of all cancer cases, and the second cause of cancer-related death [2]. A better understanding of CRC genomics has recently emerged, with promising therapeutic implications [3]. Emerging evidence suggests that the development of high-precision non-invasive screening tests using biomarkers to predict the risk of CRC or the early presence may contribute to the diagnosis and clinical practice of cancer, such as programmed cell death protein 1 (PD-1) and Na+/Ca2+ exchangers (NCXs) [3–6]. The etiology of CRC is complex, and further research is needed to elucidate the pathogenesis of CRC from various perspectives to improve the outcomes of CRC treatment.

Circular RNAs (circRNAs) are covalently closed non-coding RNA molecules, deriving from a canonical splicing mechanism [7]. CircRNAs display high extent of tissue specificity and high stability compared to linear molecules [8]. Accordingly, circRNAs are regarded as promising non-invasive biomarkers of human cancers [9]. CircRNAs have been confirmed to be involved in various signaling pathways that are crucial in human diseases [10]. Besides, circRNAs are key regulators in cancer cell proliferation, motility, apoptosis, and angiogenesis [8]. In CRC, numerous circRNAs have been identified to be deregulated, and their functions have been partly clarified [11–13]. For example, circ_0053277 was overexpressed in CRC, and its knockdown inhibited CRC cell proliferation and migration [11]. Circ_0006401 displayed high expression in CRC, and its knockdown blocked tumor cell proliferation and liver metastasis in vivo [12]. Nonetheless, there are still numerous circRNAs with unclear functions. To date, numerous differentially expressed genes (DEGs) can be acquired from Gene Expression Omnibus (GEO) database or TCGA database, and various models used for the prognostic prediction of CRC were established based on these DEGs [14–18]. A dataset (GSE77661) from GEO database provides numerous circRNAs with aberrant expression in diverse cancer tissues. Among these circRNAs, circ_0026416 was shown to have aberrantly upregulated in CRC tissues. It is of great importance to explore the role of circ_0026416 in CRC, and the molecular mechanism of circ_0026416 in CRC needs further investigation.

The molecular sponge effects of circRNA on microRNAs (miRNAs) are widely demonstrated to illustrate the functional mode of circRNAs in biological processes [19]. For example, miR-132-3p was a tumor suppressor in CRC, and circ_0020397 aggravated CRC cell malignant properties via acting as miR-132-3p sponge to sequester miR-132-3p expression [20]. By bioinformatics analysis, we screened the target miRNAs of circ_0026416 and found that miR-545-3p was previously shown to inhibit cell proliferation in CRC [21]. Therefore, we determined the relationship between circ_0026416 and miR-545-3p in CRC development. MiRNAs are well-recognized to regulate gene expression by binding to gene 3′ untranslated region (3′UTR) [22, 23]. Myosin VI (MYO6) was predicted as a target gene of miR-545-3p, and the oncogenic effect of MYO6 in CRC was widely documented [24, 25]. Thus, we explored the interaction between miR-545-3p and MYO6 in CRC to further disclose the functional mechanism of circ_0026416 in CRC.

The present study investigated the detailed functions of circ_0026416 in CRC cells and mouse models in vivo. Importantly, this study proposed a new network, circ_0026416/miR-545-3p/MYO6, to partly clarify the mechanism of circ_0026416 function in CRC, aiming to provide a theoretical basis for circ_0026416 as a biomarker in CRC.

## Materials and methods

### Specimen collection

Tumor tissues and corresponding normal (non-tumor) tissues were collected from a total of 43 CRC patients who underwent surgery at the Second Affiliated Hospital of Shandong University of Traditional Chinese Medicine. Patients never received any preoperative treatments. The characteristics of tumor tissues and normal tissues were identified by histopathology, and the clinicopathologic characteristics of these patients and the correlation between circ_0026416 expression and these characteristics were shown in Table 1. The collection of specimens was approved by all patients with written informed consent. Specimens were frozen in liquid nitrogen and placed in − 80 °C conditions. This study was permitted by the Ethics Committee of the Second Affiliated Hospital of Shandong University of Traditional Chinese Medicine.

**Table 1:** Association between circ_0026416 expression and clinical clinicopathological parameters of CRC

Parameter	Total case (*n* = 43)	Circ_0026416 expression		*P* value^a^
		Low (*n* = 21)	High (*n* = 22)	
Age (years)				0.4503
≤60	20	11	9	
>60	23	10	13	
Gender				0.6497
Female	22	10	12	
Male	21	11	10	
Tumor size				0.0003*
≤5 cm	20	16	4	
>5 cm	23	6	17	
TNM stages				0.0014*
I-II	20	15	5	
III-IV	23	6	17	
Lymphatic metastasis				<0.0001*
Negative	20	17	3	
Positive	23	4	19	

*CRC* Colorectal cancer, *TNM* tumor-node-metastasis; *P < 0.05; ^a^Chi-square test

### Cell lines

HCT116 (Bena, Beijing, China), DLD-1 (Bena), and HCT-8 (Bena) were cultured in RPMI-1640 medium (GIBCO, Grand Island, NY, USA) supplemented with 10% FBS. SW480 (Bena) was cultured in DMEM (GIBCO) containing 10% FBS. Human normal colonic epithelial cell (NCM460; Bluefbio, Shanghai, China), used as a non-cancer control, was cultured in DMEM plus 10% FBS. Human umbilical vein endothelial cells (HUVECs; Bena) were cultured in Ham’s F-12 K medium (GIBCO) containing 10% FBS. These cells were maintained in a cell incubator at 37 °C supplied with 5% CO_2_.

### Quantitative real-time PCR (qPCR)

Trizol reagent (Solarbio, Beijing, China) was utilized for total RNA isolation. For cDNA synthesis, a kit from Vazyme (Nanjing, China) named HiScript III 1st Strand cDNA Synthesis Kit, and a kit named miRNA 1st Strand cDNA Synthesis Kit (Vazyme) were applied using total RNA. Subsequently, AceQ Universal SYBR qPCR Master Mix (Vazyme) was used for qPCR reaction. Relative expression was normalized using the 2^−ΔΔCt^ method, with GAPDH (for circRNA and mRNA) or U6 (for miRNA) as the internal reference. The sequences of primers were exhibited below:

circ_0026416, F 5′-GATGAATGTCAAGCTGGCCC-3′, and R 5′-TCATGTAGGCAGCATCCACA-3′; KRT6C, F 5′-CTTCCCTGCTCTCCGAGGTA-3′, and R 5′-TGCTCAGATGGGGCAGGTAT-3′; miR-545-3p, F 5′-GCGCGTCAGCAAACATTTATT-3′, and R 5′-AGTGCAGGGTCCGAGGTATT-3′; MYO6, F 5′-AGAATCAGGAGCCGGCAAAA-3′, and R 5′-CCAAAGGCTTCTAGGAGTGGG-3′; GAPDH, F 5′-ACAGCCTCAAGATCATCAGC-3′, and R 5′-GGTCATGAGTCCTTCCACGAT-3′; U6, F 5′-CTCGCTTCGGCAGCACATATACT-3′ and R 5′-ACGCTTCACGAATTTGCGTGTC-3′.

### RNase R digestion and ActD treatment

To ensure the existence of circ_0026416, total RNA was isolated and digested with RNase R (2 U/μg; Epicentre, Madison, WI, USA) for 30 min at 37 °C and next used for qPCR.

To check the stability of circ_0026416, Actinomycin D (10 μg/mL; Sigma-Aldrich, St. Louis, MO, USA) was added into culture medium to culture cells for the indicated time (0, 4, 8, 12, or 24 h). RNA isolation was performed using these cells at different time, and subsequent qPCR was conducted.

### Cell transfection

Lipofectamine 3000 reagent (Invitrogen, Carlsbad, CA, USA) was used for cell transfection. Simply put, cells were plated into 6-well plates (5 × 10^5^ cells/well) and then transfected with oligonucleotides (50 nM) or vectors (2 μg). Small interference RNA (siRNA) targeting circ_0026416 (si-circ_0026416) and its negative control (si-NC) were synthesized by Genepharma (Shanghai, China). MiR-545-3p mimic (miR-545-3p) and miR-545-3p inhibitor (anti-miR-545-3p) and their negative controls (miR-NC and anti-miR-NC) were provided by Ribobio (Guangzhou, China). MYO6 overexpression vector (pcDNA-MYO6) and its blank control (pcDNA-con) were constructed by Genepharma.

### CCK-8 assay

After transfection, cells were plated in 96-well plates (5000 cells/well), with one sample in triplicate. Then, cells were continued to culture. CCK-8 reagent was used to treat cells at 0, 24, 48, or 72 h for 2 h. Subsequently, the absorbance at 450 nm was measured using a microplate reader (BioTek, Winooski, Vermont, USA).

### Colony formation assay

After transfection, cells were plated in 6-well plates (200 cells/well). Cells were cultured in 37 °C incubator containing 5% CO_2_ for 12 days. The growth of colonies was observed every day. The surface of cell colonies was washed by PBS, fixed by methanol and stained with crystal violet. The number of colonies was observed using a microscope (Leika, Wetzlar, Germany).

### Transwell assay

Transwell chambers coated with or without Matrigel (BD Biosciences, Franklin Lakes, NJ, USA) were used for cell invasion or cell migration analysis. At 24 h post-transfection, cells were collected and resuspended in serum-depleted culture medium. Then, cells were transferred to the top of chambers, with culture medium supplemented with 10% FBS in the bottom of chambers. Cells in chambers were cultured for 24 h to induce invasion or migration. Next, cells in the lower surface of chambers were fixed with methanol and stained with crystal violet. The magnification of 100× was used to capture the images of migration or invaded cells under a microscope.

### Tube formation assay

Ninety-six-well plates were coated with Matrigel (60 μL/well) and maintained at 37 °C incubator for 1 h. At 48 h post-transfection, tumor-conditioned medium (tumor supernatant: DMEM: FBS = 4:5:1) was prepared. HUVECs were cultured with tumor-conditioned medium and HUVEC suspension for 6 h. Five randomly selected fields were used to capture images of tube using an inverted microscope (magnification, × 40).

### Flow cytometry assay

At 48 h post-transfection, the Annexin V-FITC Apoptosis Detection Kit (Beyotime, Shanghai, China) was used to monitor cell apoptosis using a flow cytometer (BD Biosciences) according to the protocol from kit.

### Western blot

Western blot was carried out as previously mentioned [24]. All antibodies were obtained from Abcam (Cambridge, MA, USA), including anti-E-Cadherin (ab40772; dilution: 1/10,000), anti-vimentin (ab92547; dilution: 1/2,000), anti-N-Cadherin (ab76011; dilution: 1/10,000), anti-MYO6 (ab230478; dilution: 1/1,000), and goat-anti rabbit secondary antibody (ab205718; dilution: 1/20,000).

### Xenograft model

Lentivirus-packaged shRNA targeting circ_0026416 (sh-circ_0026416) and sh-NC were provided by Genepharma. Nude mice (female, 6-week-old) were purchased from Vital River Animal Technology Co., Ltd. (Beijing, China) and divided into 2 groups (*n* = 6 per group). SW480 cells were infected with lentivirus-packaged sh-circ_0026416 or sh-NC and subcutaneously injected into nude mice (2 × 10^6^ cells/mouse). The condition of mice was observed every day. Tumor volume (length×width^2^ × 0.5) was recorded once a week. After 4 weeks, all mice were euthanized to remove tumor tissues. The study of animal was approved by the Animal Care and Use Committee of the Second Affiliated Hospital of Shandong University of Traditional Chinese Medicine.

### Dual-luciferase reporter assay

The binding site between circ_0026416 and miR-545-3p was provided by circRNA interactome (https://circinteractome.nia.nih.gov/). The binding site between miR-545-3p and MYO6 3′UTR was provided by Targetscan (http://www.targetscan.org/vert_72/). The mutant-type sequences of circ_0026416 and MYO6 3′UTR (mutation at miR-545-3p binding site) were synthesized. Reporter plasmids, including circ_0026416 WT, circ_0026416 MUT, MYO6 3′UTR WT and MYO6 3′UTR MUT, were constructed into pmirGLO vector (Promega, Madison, WI, USA). HCT-8 and SW480 cells were transfected with miR-545-3p or miR-NC and reporter plasmid and next incubated for 48 h. Cells were collected and used for luciferase activity analysis using the Dual-Luciferase Reporter Assay System (Promega).

### RIP assay

Magna RIP™ Kit (Millipore, Billerica, MA, USA) was utilized for RIP assay. In brief, cells were lysed using RIP lysis solution, and cell lysates were incubated with protein A/G magnetic beads conjugated with Ago2 antibody (Millipore) or IgG antibody (Millipore). RNA complexes bound to beads were eluted and analyzed by qPCR.

### Statistical analysis

Data were collected from at least three separate experiments. The results were expressed as the mean ± standard deviation (SD). Differences in various groups were assessed by Student’s *t*-test or analysis of variance. Statistical analysis was conducted using GraphPad Prism 6 (GraphPad Software, San Diego, USA), and figures were subsequently generated. *P* less than 0.05 was considered statistically significant.

## Results

### The expression of circ_0026416 was increased in CRC tumor tissues and cells

**Fig. 1:**
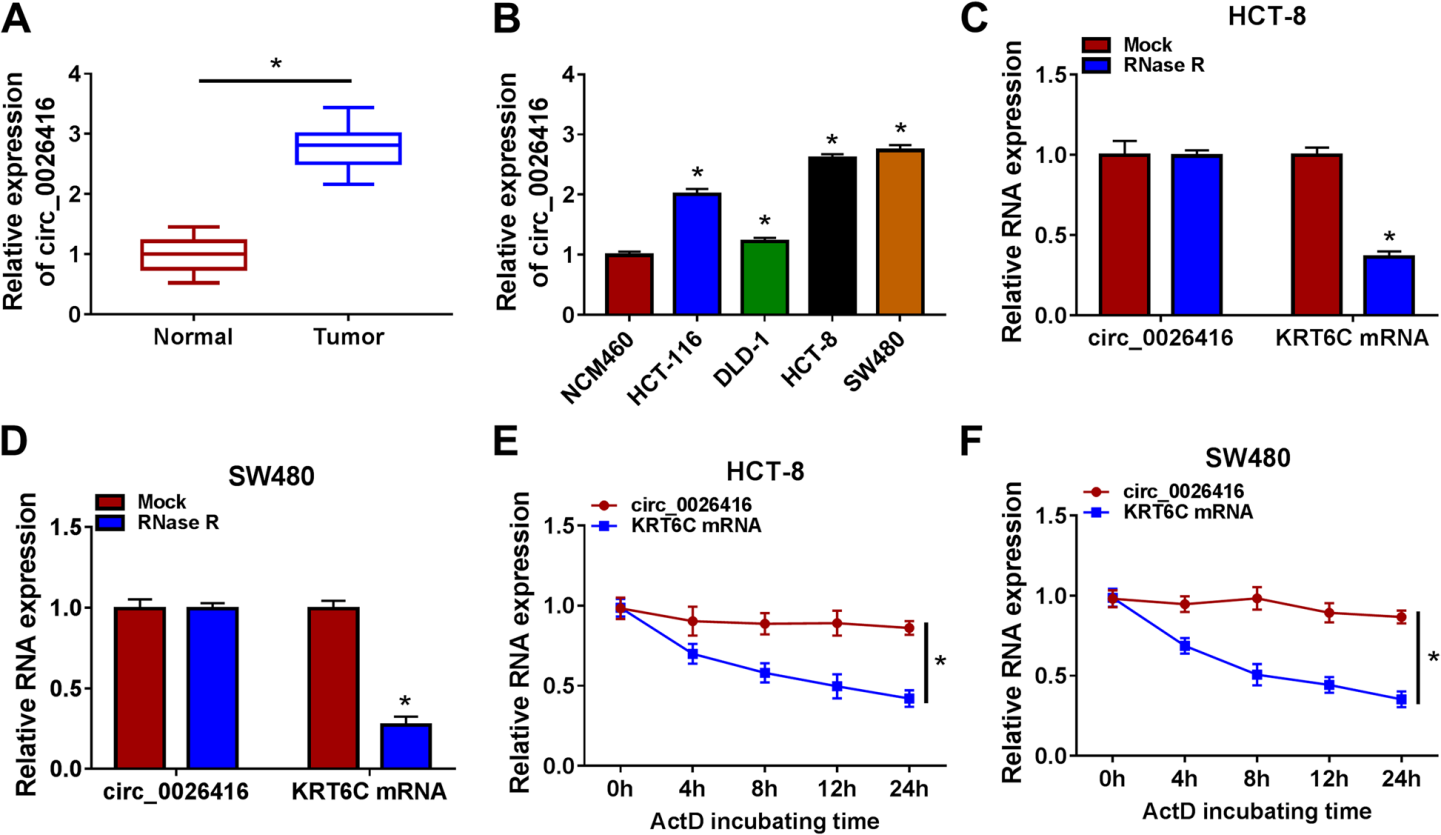
Circ_0026416 was upregulated in CRC, with high stability. **A** Circ_0026416 expression in clinical specimens was checked by qPCR. **B** Circ_0026416 expression in CRC cell lines and non-cancer cell line was checked by qPCR. **C**, **D** The existence of circ_0026416 was identified using RNase R. **E**, **F** The stability of circ_0026416 was identified using ActD. *P < 0.05

The clinical data in Table 1 displayed that high circ_0026416 expression was significantly associated with large tumor size, advanced TNM stages, and positive lymphatic metastasis. We examined the expression of circ_0026416 in clinical specimens and found that circ_0026416 expression was markedly enhanced in tumor tissues compared to normal tissues (Fig. 1A). Besides, circ_0026416 expression was also increased in CRC cell lines, including HCT-116, DLD-1, HCT-8, and SW480 cells, particularly in HCT-8 and SW480 cells (Fig. 1B). HCT-8 and SW480 cells were thus selected in the following assays. For the identification of circ_0026416 existence and stability, we found that circ_0026416 was resistant to RNase R digestion, while KRT6C mRNA was notably digested by RNase R (Fig. 1C and D). In addition, after ActD treatment, the expression of circ_0026416 was hardly declined, while the expression of KRT6C mRNA was markedly reduced (Fig. 1E, F). These data verified the existence and stability of circ_0026416 and presented the high expression of circ_0026416 in CRC tissues and cells.

### Circ_0026416 downregulation suppressed CRC cell malignant behaviors and solid tumor growth in vivo

**Fig. 2:**
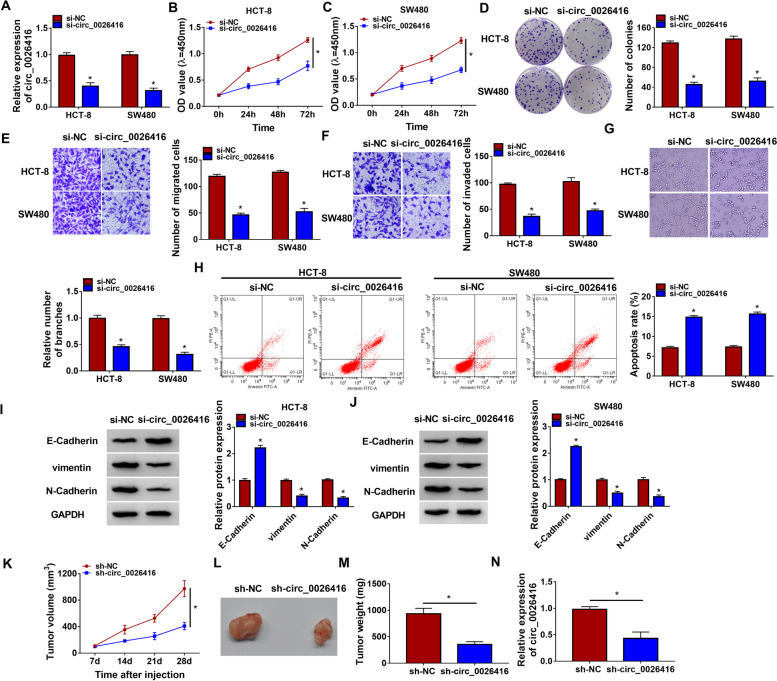
Circ_0026416 downregulation inhibited CRC development in vitro and in vivo. **A** The expression of circ_0026416 in HCT-8 and SW480 cells after si-circ_0026416 transfection was checked by qPCR. **B**, **C** Cell proliferation was checked by CCK-8 assay. **D** Cell proliferation was also checked by colony formation assay. **E**, **F** Cell migration and cell invasion were examined using Transwell assay. **G** The ability of angiogenesis was checked by tube formation assay. **H** Cell apoptosis was examined using flow cytometry assay. **I**, **J** The protein levels of E-Cadherin, vimentin, and N-Cadherin were determined by western blot. **K**–**M** Animal study was performed to determine the role of circ_0026416 knockdown in vivo. **N** The expression of circ_0026416 in tumor tissues from animal model was checked by qPCR. *P < 0.05

HCT-8 and SW480 cells were transfected with si-circ_0026416 or si-NC for functional analysis. Three different si-circ_0026416 oligonucleotides were synthesized to mediate circ_0026416 knockdown. The expression of circ_0026416 was strikingly decreased in HCT-8 and SW480 cells after si-circ_0026416 (or si-circ_0026416#1, si-circ_0026416#2) transfection (Fig. 2A; Figure S1A). In function, circ_0026416 downregulation suppressed the proliferative capacity and colony formation ability of HCT-8 and SW480 cells by CCK-8 and colony formation assays (Fig. 2B-D; Figure S1B-D). Transwell assay indicated that the migration and invasion abilities were markedly suppressed in HCT-8 and SW480 cells transfected with si-circ_0026416 (Fig. 2E, F; Figure S1E and F). Tube formation assay showed that the downregulation of circ_0026416 inhibited the number of branches of tubes, thus inhibiting angiogenesis (Fig. 2G; Figure S1G). Flow cytometry assay revealed that the apoptotic rate of HCT-8 and SW480 cells transfected with si-circ_0026416 was strikingly promoted (Fig. 2H; Figure S1H). Moreover, EMT-related markers were also quantified by western blot. The data showed that the level of E-Cadherin was enhanced, while the levels of vimentin and N-Cadherin were reduced in HCT-8 and SW480 cells transfected with si-circ_0026416 (Fig. 2I, J; Figure S1I and J). Animal study was further conducted to determine the role of circ_0026416 in vivo. The data presented that circ_0026416 knockdown in SW480 cells markedly depleted tumor volume and tumor weight (Fig. 2K–M). The expression of circ_0026416 was strikingly decreased in sh-circ_0026416-treated tumor tissues (Fig. 2N). These data indicated that circ_0026416 downregulation suppressed CRC cell malignant behaviors and solid tumor growth in vivo.

### MiR-545-3p was a target of circ_0026416

**Fig. 3:**
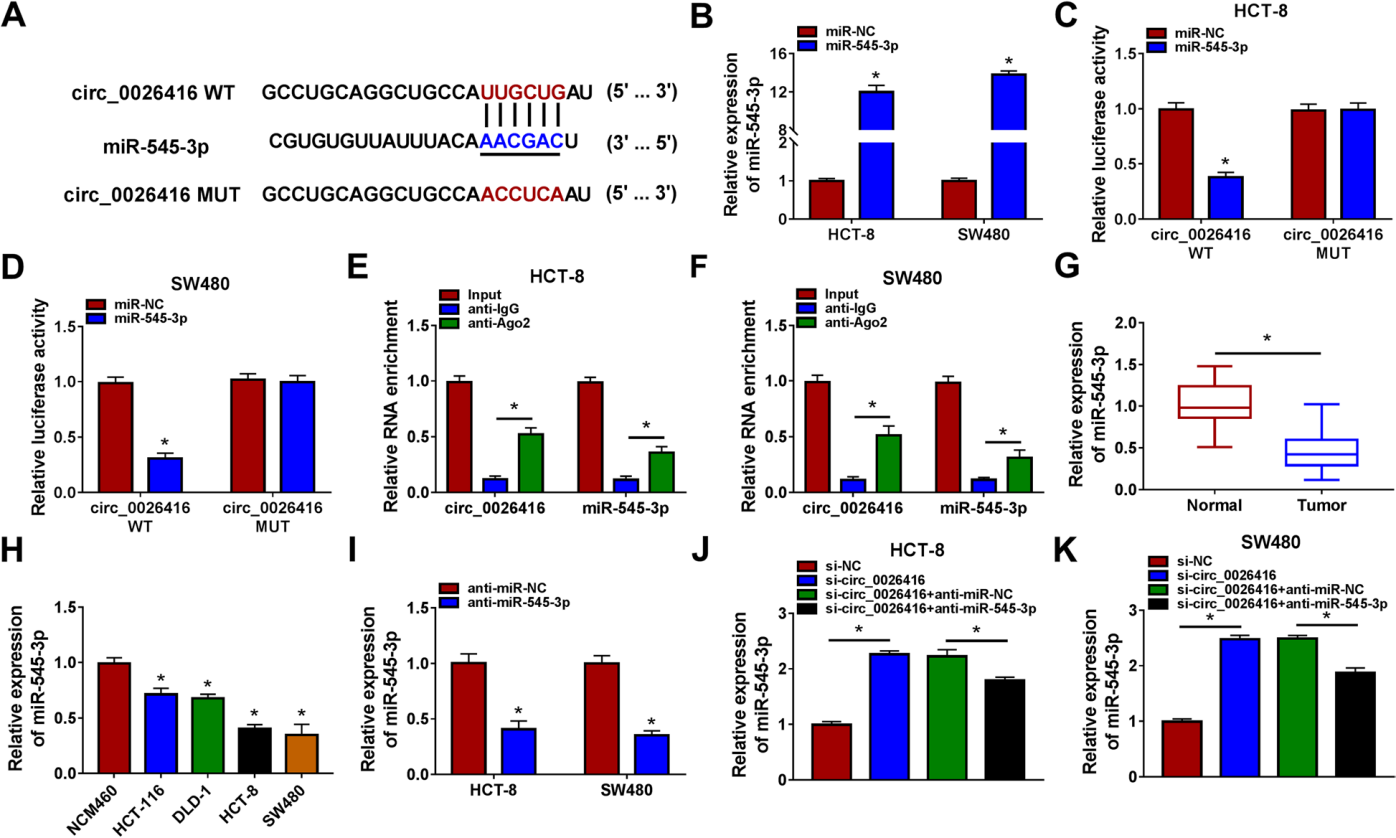
MiR-545-3p was a target of circ_0026416. **A** The binding site between miR-545-3p and circ_0026416 was provided by circRNA interactome. **B** The efficiency of miR-545-3p mimic was verified. **C**, **D** The relationship between miR-545-3p and circ_0026416 was confirmed by dual-luciferase reporter assay. **E**, **F** The relationship between miR-545-3p and circ_0026416 was confirmed by RIP assay. **G**, **H** MiR-545-3p expression in clinical specimens and cell lines was checked by qPCR. **I** The efficiency of miR-545-3p inhibitor was verified. **J**, **K** The expression of miR-545-3p in HCT-8 and SW480 cells transfected with si-circ_0026416 or si-circ_0026416 + anti-miR-545-3p was checked by qPCR. *P < 0.05

Bioinformatics tool circular RNA interactome showed the binding site between circ_0026416 and miR-545-3p sequence fragments (Fig. 3A). The expression of miR-545-3p was notably increased in HCT-8 and SW480 cells transfected with miR-545-3p mimic compared to miR-NC (Fig. 3B). Dual-luciferase reporter assay presented that miR-545-3p transfected with circ_0026416 WT significantly reduced luciferase activity in HCT-8 and SW480 cells (Fig. 3C, D). RIP assay presented that circ_0026416 and miR-545-3p were abundantly enriched in anti-Ago2 RIP group compared to anti-IgG RIP group (Fig. 3E, F). The expression of miR-545-3p was remarkably downregulated in tumor tissues and cell lines of CRC (Fig. 3G, H). Besides, miR-545-3p expression in tumor tissues was negatively correlated with circ_0026416 expression (*r* = − 0.5758, *P* < 0.0001; Figure S2). The expression of miR-545-3p was strikingly decreased in HCT-8 and SW480 cells transfected with anti-miR-545-3p (Fig. 3I). Moreover, miR-545-3p expression was markedly increased in HCT-8 and SW480 cells transfected with si-circ_0026416 but partly repressed in HCT-8 and SW480 cells transfected with si-circ_0026416 + anti-miR-545-3p (Fig. 3J, K).

### Circ_0026416 downregulation suppressed CRC cell malignant behaviors by enriching miR-545-3p

**Fig. 4:**
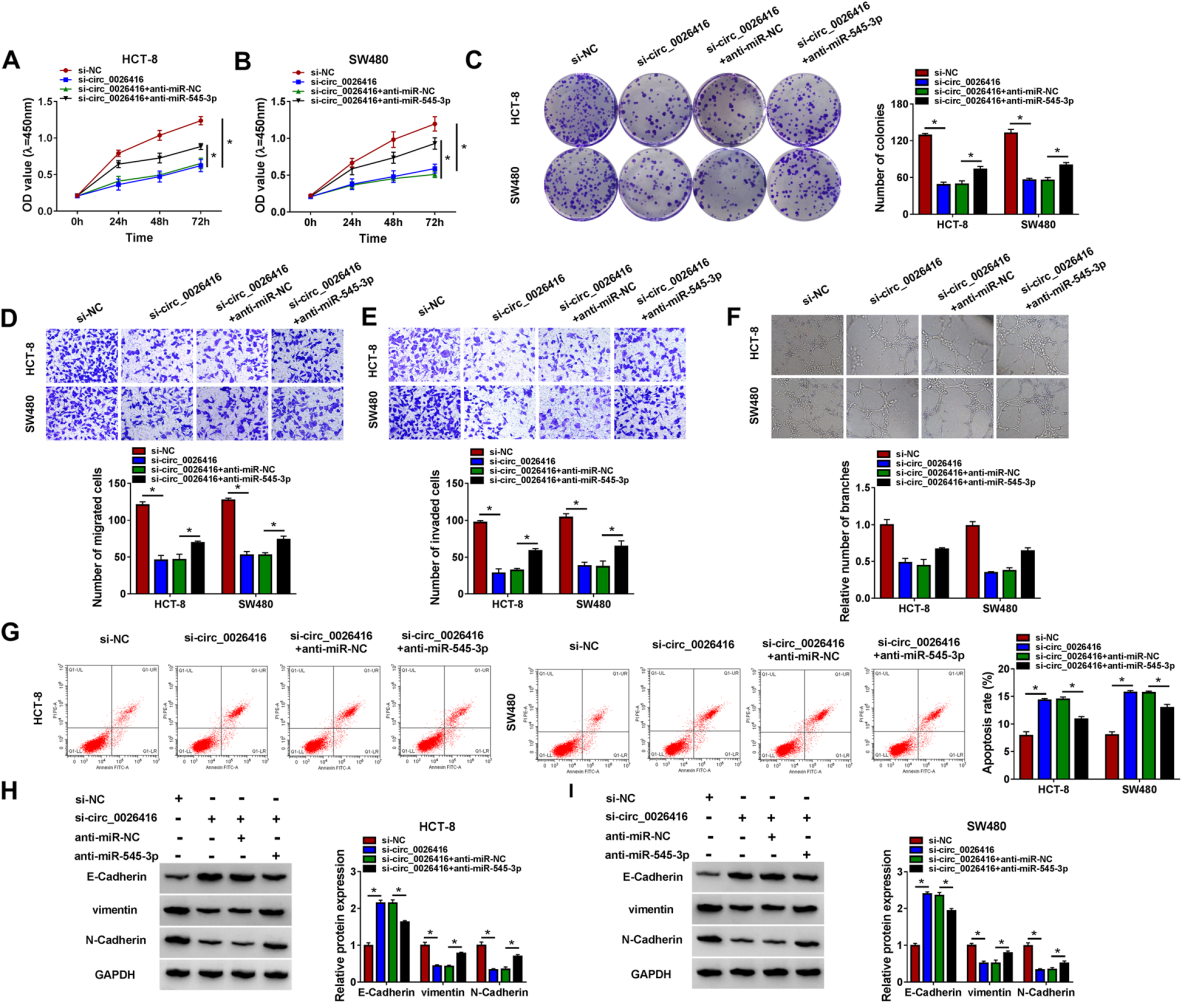
Circ_0026416 knockdown inhibited CRC cell development by enriching miR-545-3p. In function rescue experiments, **A**, **B** cell proliferation was assessed by CCK-8 assay. **C** Colony formation assay was performed to monitor cell proliferation. **D**, **E** Cell migration and cell invasion were determined by Transwell assay. **F** The ability of angiogenesis was checked by tube formation assay. **G** Flow cytometry assay was conducted to assess cell apoptosis. **H**, **I** The protein levels of E-Cadherin, vimentin, and N-Cadherin were quantified by western blot. *P < 0.05

Function rescue experiments were performed to determine the relationship between circ_0026416 and miR-545-3p. CCK-8 assay and colony formation assay presented that cell proliferation and colony formation were suppressed in HCT-8 and SW480 cells transfected with si-circ_0026416 but partly recovered in cells transfected with si-circ_0026416 + anti-miR-545-3p (Fig. 4A–C). Transwell assay manifested that cell migration and cell invasion were blocked in HCT-8 and SW480 cells after circ_0026416 knockdown, and further miR-545-3p downregulation partly enhanced cell migration and invasion (Fig. 4D, E). Tube formation assay revealed that the inhibition of miR-545-3p promoted angiogenesis that was inhibited by circ_0026416 knockdown alone (Fig. 4F). In addition, circ_0026416 knockdown-induced HCT-8 and SW480 cell apoptosis was largely alleviated by miR-545-3p knockdown (Fig. 4G). Moreover, E-Cadherin level promoted in HCT-8 and SW480 cells transfected with si-circ_0026416 was partly repressed in HCT-8 and SW480 cells transfected with si-circ_0026416 + anti-miR-545-3p, while vimentin and N-Cadherin levels suppressed in HCT-8 and SW480 cells transfected with si-circ_0026416 were partly recovered in HCT-8 and SW480 cells transfected with si-circ_0026416 + anti-miR-545-3p (Fig. 4H, I). The data indicated that circ_0026416 downregulation suppressed CRC cell malignant behaviors by enriching miR-545-3p.

### MiR-545-3p suppressed MYO6 expression

**Fig. 5:**
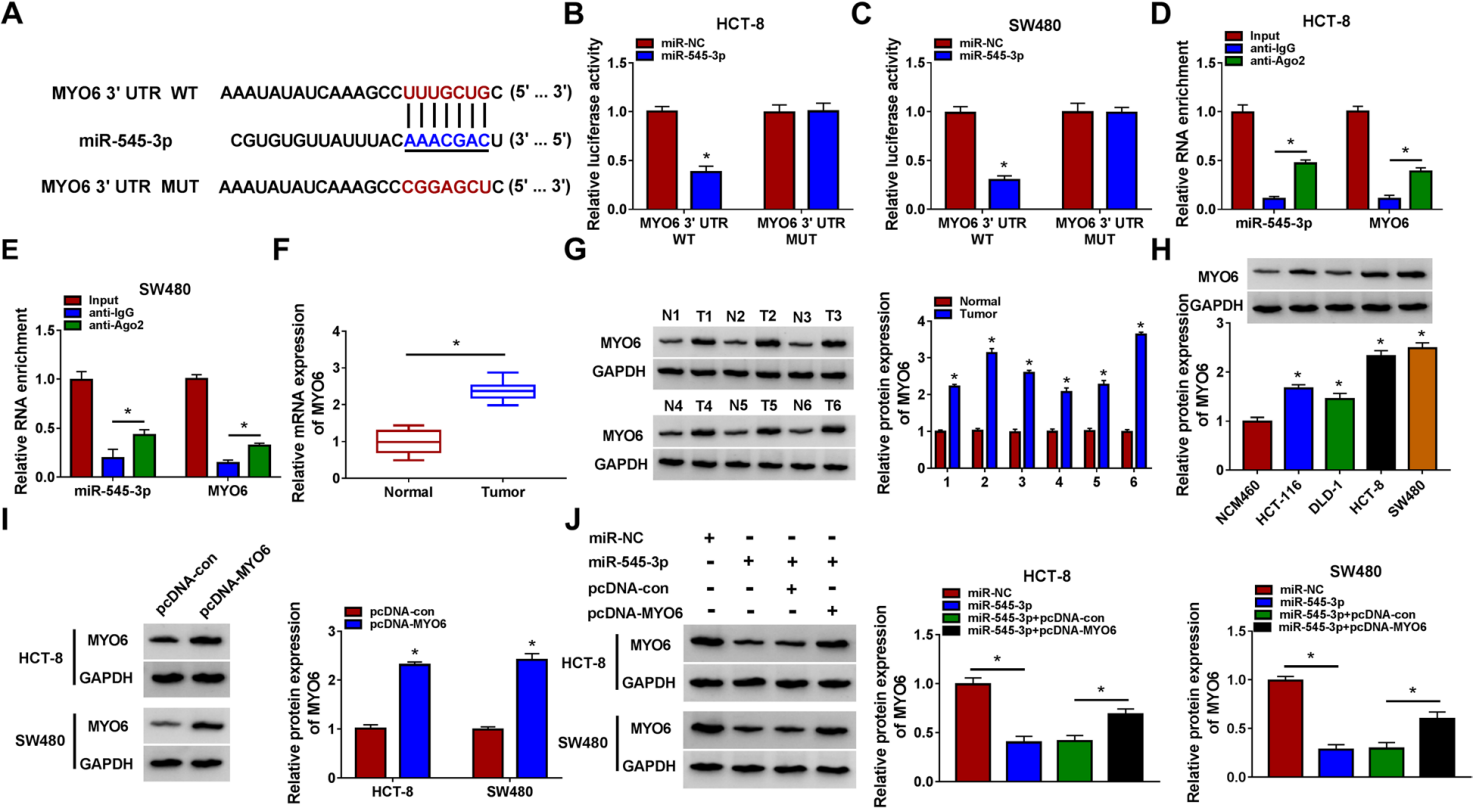
MYO6 was a target of miR-545-3p. **A** The binding site between MYO6 and miR-545-3p was provided by Targetscan. **B**, **C** The relationship between MYO6 and miR-545-3p was confirmed by dual-luciferase reporter assay. **D**, **E** The relationship between MYO6 and miR-545-3p was confirmed by RIP assay. **F**, **G** The expression of MYO6 mRNA and protein in clinical specimens was checked by qPCR and western blot. **H** The expression of MYO6 protein in CRC cell lines and non-cancer cell line was detected by western blot. **I** The expression of MYO6 protein in HCT-8 and SW480 cells transfected with pcDNA-MYO6 was checked by western blot. **J** The expression of MYO6 protein in HCT-8 and SW480 cells transfected with miR-545-3p alone or miR-545-3p + pcDNA-MYO6 was detected by western blot. *P < 0.05

Bioinformatics tool Targetscan showed the binding site between miR-545-3p and MYO6 3′UTR, implying that MYO6 was a potential target of miR-545-3p (Fig. 5A). Dual-luciferase reporter assay showed that miR-545-3p transfected with MYO6 3′UTR significantly reduced luciferase activity in HCT-8 and SW480 cells (Fig. 5B, C). RIP assay displayed that miR-545-3p and MYO6 were markedly enriched in the anti-Ago2 RIP group compared to anti-IgG RIP group (Fig. 5D, E). The expression of MYO6 mRNA was notably reinforced in tumor tissues (Fig. 5F), and the protein level of MYO6 was also notably reinforced in 6 randomly selected tumor tissues from CRC patients (Fig. 5G). The protein level of MYO6 was markedly enhanced in CRC cell lines (Fig. 5H). In addition, the protein level of MYO6 was largely increased in HCT-8 and SW480 cells after pcDNA-MYO6 transfection (Fig. 5I). The protein level of MYO6 was significantly declined in HCT-8 and SW480 cells transfected with miR-545-3p alone but recovered in cells transfected with miR-545-3p + pcDNA-MYO6 (Fig. 5J). The data mainly indicated that miR-545-3p targeted MYO6 and inhibited MYO6 expression.

### MiR-545-3p restoration inhibited CRC cell malignant behaviors by inhibiting MYO6 expression

**Fig. 6:**
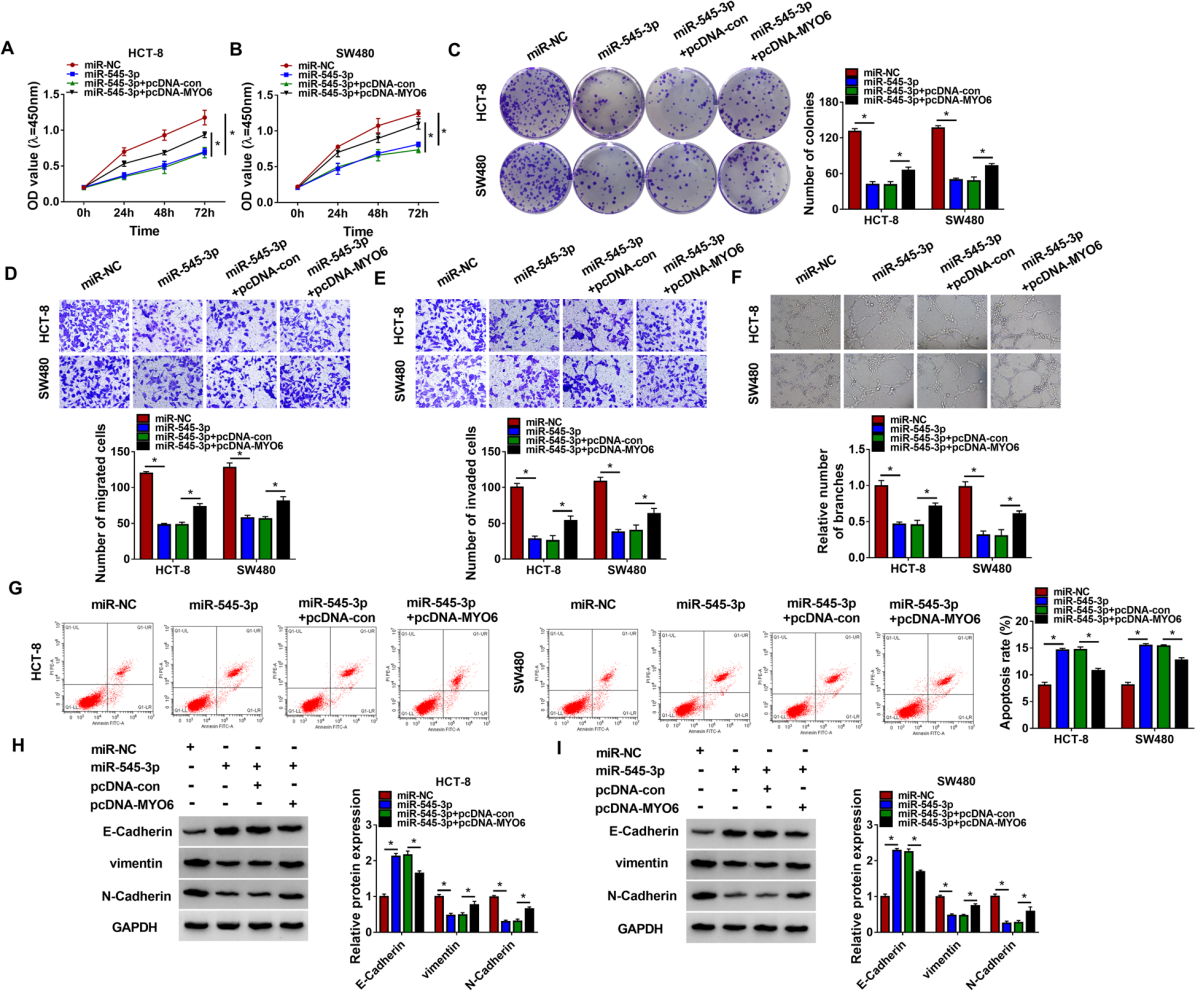
MiR-545-3p restoration blocked CRC cell malignant behaviors by targeting MYO6. In function rescue experiments, **A**, **B** cell proliferation was assessed by CCK-8 assay. **C** Cell proliferation was assessed by colony formation assay. **D**, **E** Cell migration and cell invasion were examined using Transwell assay. **F** The ability of angiogenesis was checked by tube formation assay. **G** Flow cytometry assay was conducted to assess cell apoptosis. **H**, **I** The protein levels of E-Cadherin, vimentin and N-Cadherin were quantified by western blot. *P < 0.05

CCK-8 assay and colony formation assay manifested that cell proliferative capacity and colony formation ability were notably inhibited in HCT-8 and SW480 cells transfected with miR-545-3p alone but partly recovered in cells transfected with miR-545-3p + pcDNA-MYO6 (Fig. 6A–C). Transwell assay presented that the capacity of cell migration and cell invasion was notably sequestered in HCT-8 and SW480 cells transfected with miR-545-3p alone but partly restored in cells transfected with miR-545-3p + pcDNA-MYO6 (Fig. 6D, E). Tube formation assay indicated that angiogenesis in HCT-8 and SW480 cells was inhibited by miR-545-3p restoration but partially recovered by MYO6 reintroduction (Fig. 6F). Flow cytometry assay revealed that miR-545-3p restoration-induced HCT-8 and SW480 cell apoptosis was relieved by the reintroduction of MYO6 (Fig. 6G). Additionally, the expression of E-Cadherin was increased, while the expression of vimentin and N-Cadherin was decreased in HCT-8 and SW480 cells transfected with miR-545-3p alone. However, the expression of E-Cadherin was partly repressed, while the expression of vimentin and N-Cadherin was restored in HCT-8 and SW480 cells transfected with miR-545-3p + pcDNA-MYO6 (Fig. 6H, I). The data revealed that miR-545-3p restoration inhibited CRC cell malignant behaviors by inhibiting MYO6 expression.

### Circ_0026416 knockdown enriched miR-545-3p expression and thus inhibited MYO6 expression

**Fig. 7:**
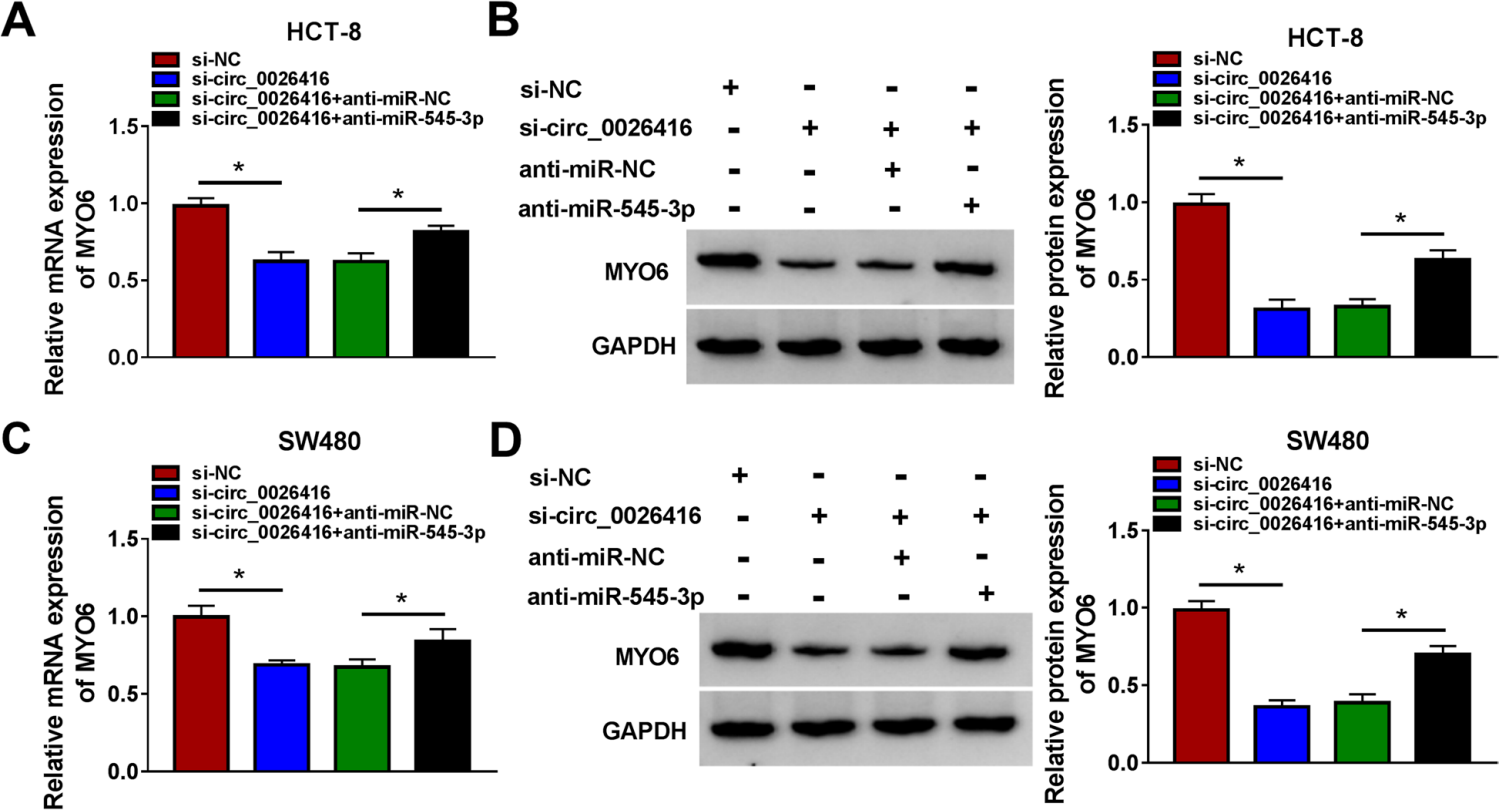
Circ_0026416 indirectly regulated MYO6 expression by targeting miR-545-3p. **A**, **B** The expression of MYO6 mRNA and protein in HCT-8 cells transfected with si-circ_0026416 or si-circ_0026416 + anti-miR-545-3p was detected by qPCR and western blot. **C**, **D** The expression of MYO6 mRNA and protein in SW480 cells transfected with si-circ_0026416 or si-circ_0026416 + anti-miR-545-3p was detected by qPCR and western blot. *P < 0.05

We further examined the expression of MYO6 mRNA and protein in HCT-8 and SW480 cells transfected with si-circ_0026416 alone or si-circ_0026416 + anti-miR-545-3p. The data showed that the mRNA and protein levels of MYO6 were strikingly decreased in HCT-8 and SW480 cells transfected with si-circ_0026416 alone but largely recovered in cells transfected with si-circ_0026416 + anti-miR-545-3p (Fig. 7A-D). The data indicated that circ_0026416 knockdown enriched miR-545-3p expression and thus inhibited MYO6 expression.

## Discussion

Recently, the function of newly identified circRNAs in CRC has been declared by an increasing number of literature [26–28], which contributes to the understanding of CRC pathogenesis. Our study exploited the function of circ_0026416 whose expression was strengthened in CRC tissues and cells and found that circ_0026416 silencing inhibited CRC cell proliferation, migration, invasion, angiogenesis, and survival. In terms of mechanism, we identified that circ_0026416 was involved in CRC development by regulating MYO6 expression via acting as miR-545-3p sponge. These results deepened our understanding of the role of circRNA in CRC progression.

Due to the advance of high-throughput RNA sequencing, a large number of circRNAs are considered to play important effects in cancer progression [29]. Based on this technology, circ_0026416 was identified to be markedly upregulated in CRC tumor tissues in GEO database (accession: GSE77661). A recent study reported that circ_0026416 expression was abnormally reinforced in tissues and plasma of CRC patients, and high circ_0026416 expression was linked to poor prognosis of CRC patients [30]. Further study manifested that the deficiency of circ_0026416 inhibited CRC cell proliferation, invasion, and solid tumor growth in vivo [30]. Consistent with this study, our functional experiments displayed that circ_0026416 downregulation repressed the proliferation, migration, invasion, and angiogenesis of HCT-8 and SW480 cells and blocked solid tumor growth in animal models. These findings highlighted the cancer-promoting effects of circ_0026416 in CRC. At present, gene therapy is a promising treatment for cancer, and the delivery of siRNA-mediated oncogene suppression based on nanotechnology is a new strategy for CRC therapy [31, 32]. This study hinted that siRNA-mediated circ_0026416 inhibition might be a novel strategy to prevent the development of CRC.

To address the action mechanism of circ_0026416 in CRC, we identified miRNAs targeted by circ_0026416. MiR-545-3p attracted our interest because the role of miR-545-3p had been mentioned in CRC. For example, miR-545-3p overexpression inhibited CRC cell proliferation and colony formation, and its overexpression also impeded tumor growth in vivo [21]. Besides, miR-545-3p enrichment effectively induced CRC cell cycle arrest at G1 phase [33]. Our study discovered that miR-545-3p deficiency partly reversed the effects of circ_0026416 downregulation and recovered CRC cell malignant behaviors, while miR-545-3p restoration suppressed the growth, migration, invasion, and angiogenesis of CRC cells. Interestingly, miR-545-3p was also reported to inhibit the progression of several other cancers, such as hepatocellular carcinoma and non-small cell lung cancer [34, 35], suggesting that miR-545-3p was a widely-acknowledged tumor suppressor in cancers. Here, partial restoration was observed in rescue experiments, possibly because miR-545-3p was only one of the targets of circ_0026416, and all of these targets functioned in circ_0026416-mediated regulatory networks.

Further analysis identified that miR-545-3p inhibited MYO6 expression by binding to MYO6 3′UTR. The reintroduction of MYO6 abolished the effects of miR-545-3p restoration and restored the malignant behaviors of CRC cells. MYO6 was an oncogene, which had been demonstrated in diverse cancers [36–38]. In CRC, the knockdown of MYO6 was shown to repress CRC cell growth and promote cell apoptosis [24]. Moreover, MYO6 was proposed to be involved in the circ_0000231/miR-502-5p pathway in the regulation of CRC progression [39]. Similarly, we found that circ_0026416 downregulation reduced the expression of MYO6 via enriching the level of miR-545-3p; thus, the circ_0026416/miR-545-3p/MYO6 regulatory network was established. Our study highlighted that oncogenic MYO6 was suppressed by miR-545-3p, while circ_0026416 acted as miR-545-3p sponge to alleviate the suppression of MYO6, thus promoting CRC carcinogenesis.

This study is only a preliminary study that illustrates the role of circ_0026416 in CRC, and the clinical application of circ_0026416 needs to be further exploited. Besides, the existing evidence published that some key regulators in CRC tumorigenesis, such as epiregulin (EREG) and amphiregulin (AREG), were differently expressed in right- and left-sided cases of CRC, and the mutation rate of some oncogenes or tumor suppressor genes was various in different sidedness of CRC [40]. This suggested that the expression pattern might be different in in right- and left-sided CRC. However, there was a lack of data on the sidedness of CRC, and we thus did not assess the difference of circRNA role in right- and left-sided cases. The future work should attach importance to these issues.

## Conclusion

In summary, our study discovered that circ_0026416 downregulation inhibited CRC cell malignant behaviors in vitro and solid tumor growth in vivo. Importantly, we provided a new mechanism that circ_0026416 knockdown suppressed CRC development by depleting MYO6 via enriching miR-545-3p. We mainly defined that circ_0026416 was a carcinogenic driver in CRC, and circ_0026416 promoted CRC progression partly by targeting the miR-545-3p/MYO6 pathway. Further extensive in-depth studies are required to confirm these findings. All in all, this study hints that the inhibition of circ_0026416 mediated by siRNA may be a promising approach for CRC therapy.

## Supplementary Information

**Additional file 1: Figure S1.** Circ_0026416 downregulation inhibited CRC cell malignant behaviors. (A) The expression of circ_0026416 in HCT-8 and SW480 cells after si-circ_0026416#1 or si-circ_0026416#2 transfection was checked by qPCR. In these transfected cells, (B-D) cell proliferation was checked by CCK-8 assay and colony formation assay. (E and F) Cell migration and cell invasion were examined using Transwell assay. (G) The ability of angiogenesis was checked by tube formation assay. (H) Cell apoptosis was examined using flow cytometry assay. (I and J) The protein levels of E-Cadherin, vimentin and N-Cadherin were determined by western blot. *P<0.05.

**Additional file 2: Figure S2.** MiR-545-3p expression was negatively correlated with circ_0026416 expression in tumor tissues.

## Data Availability

Not applicable.

## Abbreviations

CRC: Colorectal cancer
circRNAs: Circular RNAs
siRNA: Small interference RNA
MYO6: Myosin VI
qPCR: Quantitative real-time PCR
EMT: Epithelial mesenchymal-transition
