# Supplementary figures and images for: Circ_0026416 downregulation blocks the development of colorectal cancer through depleting MYO6 expression by enriching miR-545-3p
Source: World J Surg Oncol. 2021 Oct 14;19:299. doi: 10.1186/s12957-021-02407-y (PMC8515727; doi:10.1186/s12957-021-02407-y)

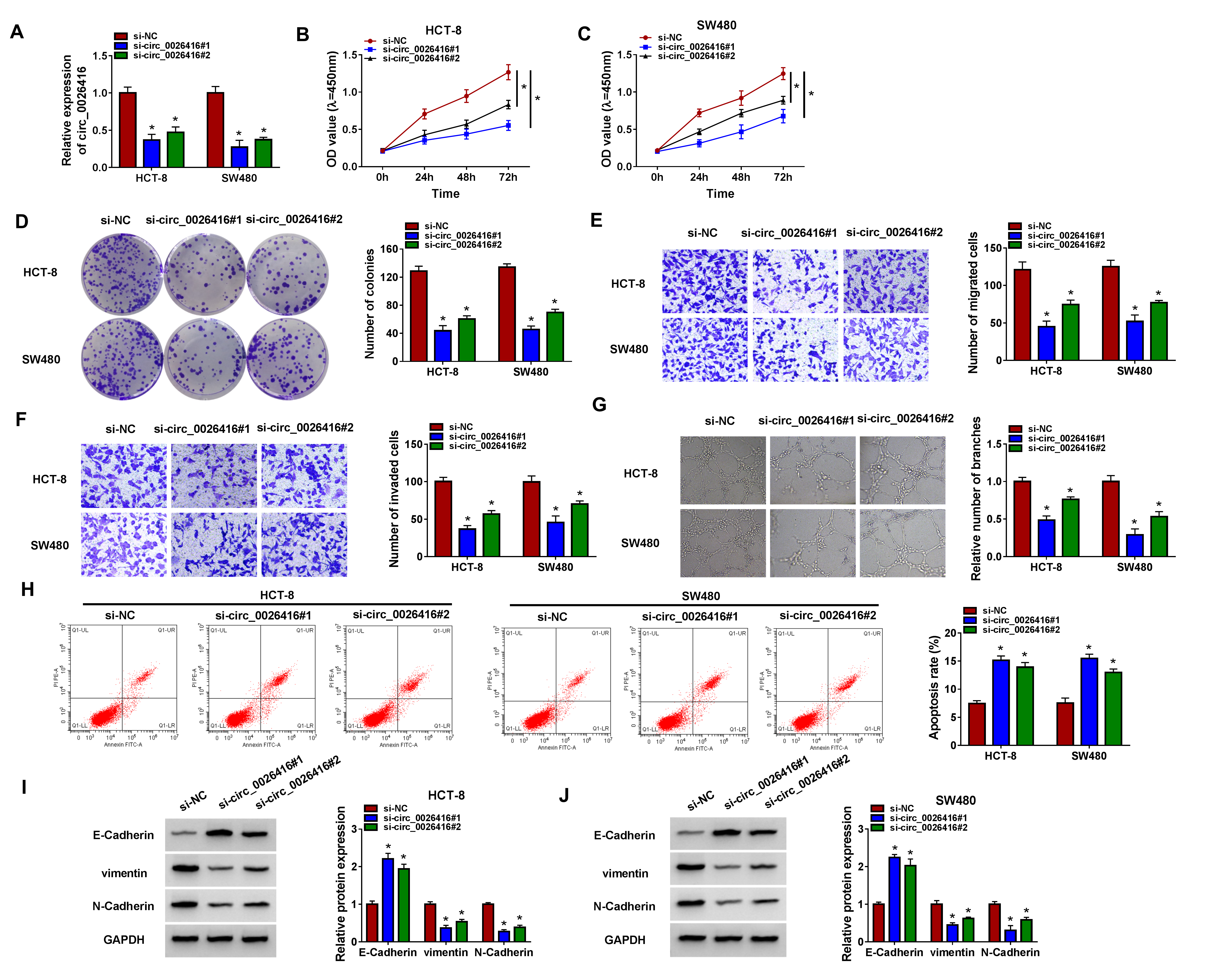

Supplement: Supplementary file 1 — Additional file 1: Figure S1. Circ_0026416 downregulation inhibited CRC cell malignant behaviors. (A) The expression of circ_0026416 in HCT-8 and SW480 cells after si-circ_0026416#1 or si-circ_0026416#2 transfection was checked by qPCR. In these transfected cells, (B-D) cell proliferation was checked by CCK-8 assay and colony formation assay. (E and F) Cell migration and cell invasion were examined using Transwell assay. (G) The ability of angiogenesis was checked by tube formation assay. (H) Cell apoptosis was examined using flow cytometry assay. (I and J) The protein levels of E-Cadherin, vimentin and N-Cadherin were determined by western blot. *P<0.05. [file 12957_2021_2407_MOESM1_ESM.tif]

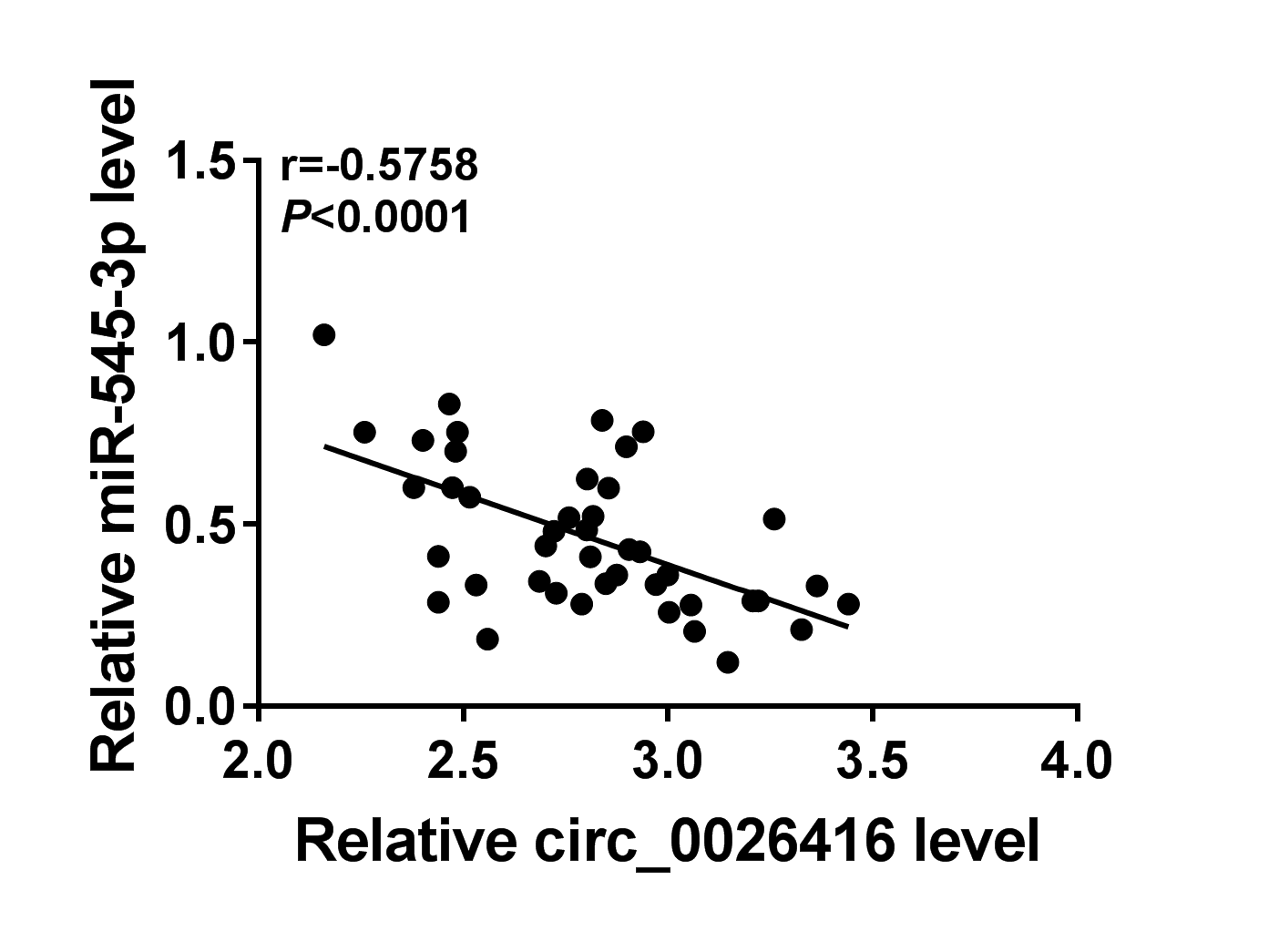

Supplement: Supplementary file 2 — Additional file 2: Figure S2. MiR-545-3p expression was negatively correlated with circ_0026416 expression in tumor tissues. [file 12957_2021_2407_MOESM2_ESM.tif]
